# Lessons on Ethical Decision Making from the Bioscience Industry

**DOI:** 10.1371/journal.pmed.0030129

**Published:** 2006-04-04

**Authors:** Jocelyn E Mackie, Andrew D Taylor, David L Finegold, Abdallah S Daar, Peter A Singer

## Abstract

Mackie and colleagues performed over 100 interviews with managers and executives at 13 bioscience companies to learn about bioindustry ethics from their perspective.

Ethical issues are a growing concern for companies, in the wake of a series of corporate governance scandals and the accompanying sharp decline in societal and investor trust in firms. Some companies have responded to these concerns by creating internal ethics programs. In the aerospace sector, for example, companies have focused these efforts on ensuring compliance with government regulations, while in the energy sector, ethics initiatives have concentrated on environmental issues and corporate and social responsibility [ 1].

Companies in pharmaceutical, biotech, and bioagricultural industries must not only comply with a wide array of government regulations and balance the profit motive with social responsibility, but also must deal with the complex array of ethical issues raised by doing business in the biosciences. These complex issues include the production and sale of genetically modified foods; gene therapy experiments and embryonic stem cell research to produce new therapies; animal testing for pharmaceuticals; drug pricing at home and in developing countries; the potential misuse of personal genetic information; how to appropriately commercialize and profit from genetic and biological samples; and the creation of transgenic animals for drug production.

Although a theoretical debate rages about whether bioethicists should consult to industry [ 2, 3], no one has systematically examined from the standpoint of bioscience companies themselves how they address these ethical issues and why they do so. In understanding the complex relationship between bioethics and industry, there is a need to obtain insight from the people closest to the phenomenon. Some researchers would like to discount the views of corporate managers, but to do so would inappropriately ignore a very legitimate viewpoint, and a good starting place to begin to understand the issues faced, and approaches taken, by companies. To address this gap, our research team sought to uncover how bioscience companies, from global corporations to small start-ups, address ethical challenges specific to bioscience firms.

Using the case study method (see Box 1), we performed more than 100 in-depth, face-to-face interviews with top managers and executives at 13 bioscience companies to learn about bioindustry ethics from their perspective (see Table 1 for a list and description of the 13 companies). Of the 13 companies, the majority can be classified as biotechnology companies, engaged in developing medical products, tools, and bioagricultural or industrial products. We also chose to include some companies for comparison that are part of the biotech value chain: a few pharmaceutical companies that often partner with biotechnology firms, and a contract research organization that is a supplier to biotech firms; thus, we use the term bioscience rather than biotech. We invited 19 companies to take part in the project, and 13 agreed (four pharmaceutical, one biotech, and one bioagricultural company declined).

**Table 1:**
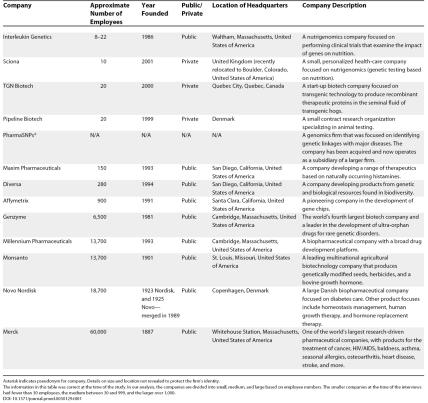
Description of Study Companies

Ethical issues are a growing concern for companies.

The companies were not approached because they were representative of the bioscience industry, but rather because we knew they had implemented interesting and varied mechanisms to address ethical decision making from which we felt the rest of the industry could learn. In our selection, we were also looking for variety in company size, type, and location. The individual company case studies have been published as a book, *BioIndustry Ethics* [ 4], and each case provides a detailed examination of the company, the ethical issues it faces, and the mechanisms the company is using to address these issues.

What was not covered in the book (and is reported in this article) are the findings from a cross-case comparative analysis of the 13 case studies. The data were analyzed under four themes. The first theme, and focus of this article, examined what mechanisms are being used by these companies to address ethical issues. This theme produced a list of mechanisms that can help to address bioindustry ethics; these are highlighted in Box 2 and described below. The second theme of our analysis was an attempt to see how the companies evaluated the effectiveness of their ethics mechanisms. As will be discussed, this theme, although important, has only been incorporated by a few companies in our study, and only on a preliminary basis, illustrating that this area requires further research and development.

Although not discussed at length in this article, the third theme was to understand the reasons why these companies have decided to address ethical issues, and the fourth theme was to learn what ethical issues these companies are facing. The results from theme three produced a list of six reasons why these companies are addressing bioindustry ethics ( Box 3), and the ethical issues facing each firm from theme four are described in Table 2.

**Table 2:**
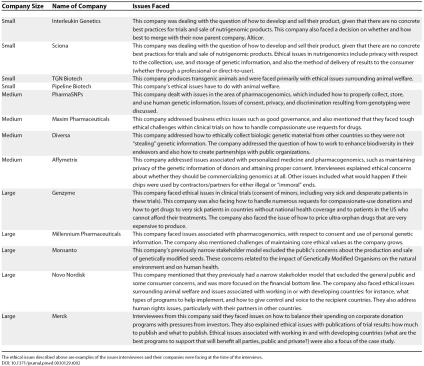
Ethical Issues Faced by Study Companies

## How Are Bioscience Companies Addressing Bioethics?

Our study revealed a variety of mechanisms that are presently being used by bioscience companies to address ethical issues. These mechanisms offer insight and provide ideas on how other bioscience companies could implement mechanisms in their firms to address their own ethical issues. The specific mechanisms fall into five mutually reinforcing approaches ( Box 2). All of our findings represent a snapshot of what these companies were doing to address bioindustry ethics at the time of our interviews.

## Approach One: Ethical Leadership

### Founder/CEO/management ethical leadership

Ethical sensitivity and behaviour often spring from a company's founders or current leadership. All 13 companies mentioned ethical leadership as a key component of their approach to ethics. For some, a CEO or an ethical leader within the company was the driving force behind their approach to ethics. At Millennium Pharmaceuticals, the CEO/Founder's commitment to ethics was the driving force behind the company's ethical culture. Their VP of Ethics and Corporate Responsibility explained: “In the business ethics arena it is particularly important…to have an identifiable leader with charisma and a deep sense of commitment to the institution and someone who models ethical behaviour for his or her employees.”

### An ethics department

As companies grow, we found that they tend to become more systematic in the way they address ethics. While some of the small- and medium-sized companies employed individuals, including key business leaders who included ethics as part of their job responsibilities, several of the larger companies we studied developed separate departments whose sole focus is to address ethical issues. Merck and Millennium both have an internal Ethics Office, where individuals with an ethical issue can go for a confidential consultation. Novo Nordisk has several divisions within the company that drive and monitor the company's Triple Bottom Line (social, environmental, financial) performance. They also have a Bioethics Director focused on addressing the company's environmental, human, and animal-related ethical issues.

## Approach Two: External Expertise

### External ethics consultant

Several of the companies used external consultants at various stages in their development to provide ethics education or expertise that was missing internally. For Sciona, which was still in its start-up phase, an external ethics consultant was its primary ethics mechanism. Sciona's consultant helped the company navigate the ethical issues associated with its business of providing genetic-based health and diet information. The consultant reviewed marketing material to make sure it was ethically appropriate for the audience and encouraged the company to re-think its direct-to-consumer sales approach and to engage in a dialogue with the United Kingdom Human Genetics Commission.

### Ethics advisory boards

Ethics Advisory Boards (EABs) were used as the primary mechanism by two of the medium-sized companies, PharmaSNPs (this company was acquired and no longer exists, and as a result, the company name has been anonymized) and Affymetrix, as a mechanism to provide independent guidance and advice on ethical issues the companies faced. The EABs were composed of outside members representing the fields of medicine, law, religion, and ethics—and at one of the companies, lay members of the general public. The members would meet on a regular basis and would discuss, debate, and provide actionable guidance on specific issues. An Affymetrix EAB member explained one of the roles of the EAB: “We hold space, a focus and a safe place for [the company] to have non-core business discussions. To ask questions like: ‘Is there anything wrong with this deal?’ or ‘How far should we go to be ethical?’ We help them clarify why a certain activity is acceptable and why other choices are not.”

## Approach Three: Internal Ethics Mechanisms

### Hiring practices focused on ethics

Some companies in our study are now putting weight on candidates' values, in addition to their past performance and technical expertise, when making hiring decisions. Six of the companies in this study include interview questions during the hiring process that aim to assess how the potential employee's values align with the ethical values of the company. For example, employees from both Millennium and Maxim explained that technical skills and experience are now combined with the candidate's behavioural and ethical fit when assessing the candidate's merits.

### Employee performance evaluations.

A key driver of employee behaviour in any organization is the types of behaviours that are rewarded and promoted by upper management. One medium-sized and three large-sized companies that were interviewed have incorporated ethics into employee performance reviews. For example, a Merck interviewee explained that the intention when designing a performance system is to not create incentives that encourage employees to bend the rules. The employee said: “We try not to put people in situations where they have to ‘make a number’ so that they won't be tempted to give a $10,000 research grant to a doctor just to make a sale and meet that number.”

### Ethics education and forums for ethics discussion

The majority of the medium- and large-sized companies we interviewed have developed formal ethics education sessions on topics such as research ethics and informed consent. Several of these firms have also introduced less formal forums for ethics discussion, where employees can voice concerns and have questions answered. At Monsanto, these are called “town hall meetings.” Millennium used popular film screenings—e.g., *Gattaca* and *Inherit the Wind*—facilitated by an outside ethics expert to draw out issues for ethics debates among employees. Millennium, Genzyme, and Merck have also implemented an Ethics Helpline that employees can call anonymously to get guidance about ethical issues.

### Ethical reinforcement techniques

All of the companies we interviewed use techniques to reinforce ethics within the company, although these techniques tend to be more formally organized in the larger firms. Some try to remind employees of the importance of ethics by defining core values as part of the company's culture (such as Genzyme's “Putting the Patient First” approach), and some provide oral and visual reinforcements (by printing them on placards around the company buildings or on employees' mouse pads, as at Millennium). Ethical guidelines in areas such as clinical trials and sales and marketing of pharmaceuticals were given during training and then reinforced with oral and visual reminders. For these techniques to be effective and to have an impact on the ethical conduct of employees, our interviewees explained that they need to be continually and consistently reinforced by management.

## Approach Four: External Ethics Engagement

### Ethics mechanisms with partners and suppliers

Of the 13 companies, seven, spanning all sizes, have extended their ethics approach to their business partners—to share the benefits created by these companies and/or to try to ensure that their partners also follow high ethical standards. The primary bioindustry ethics mechanism used by Diversa, for example, is the benefit-sharing partnerships they have developed with countries that are involved with the collection of biological samples. Instead of secretly taking genetic material from these countries, referred to as “biopiracy,” Diversa forms partnerships—with, for example, a national park—to collect and process samples. In return, the company provides its partner with some up-front funding and training, along with a royalty percentage on any discoveries that originate from the samples.

Novo Nordisk has extended its Triple Bottom Line approach beyond the company to include its suppliers, who must fill out a social/ environmental survey to assess whether they are following the same social and environmental norms to which Novo Nordisk ascribes. If a supplier is found to be violating some of these norms, Novo Nordisk will work with them to improve their standards.

### Transparent engagement mechanisms with stakeholders

Companies of all sizes in our study (seven of the 13) are engaging with external stakeholders on ethical issues, although this seemed to become more of a necessity as firms became larger and higher-profile. These stakeholders include local communities, nongovernmental organizations, governments, interest groups, and consumers. One example is Novo Nordisk's invitations to animal welfare activist groups to tour its labs and to discuss potential solutions to their differences. Explained one Novo Nordisk VP: “It was successful because of the openness and because we weren't seeking consensus. What we were seeking was to understand each other and to look for areas of commonality… However, some companies think that the dialogue is sufficient. But it's not. It requires action and responsiveness. There has to be a tangible outcome.”

Another example of listening to stakeholders and acting on stakeholder concerns includes TGN Biotech's efforts to engage citizens of a community in which the company planned to build a pig farm. They held an information night to educate the community about their science and to answer their questions. Interviewees explained that if the community had decided that it did not want the company to build the genetically modified pig farm in their community, the company was committed to finding another location.

### Transparency of science

Some of the fear in society about new science and technology stems from a perception that companies develop their science and technology secretively and do not share negative results. The Vioxx incident with Merck, which occurred after our study, demonstrates the importance of transparency. According to our findings, this is one area where companies are presently struggling to find a balance between protecting important patent and research information and the need to be transparent in a manner that will meet public satisfaction. One mechanism to address this issue was highlighted by the Director of Clinical Reporting at Novo Nordisk, who reported that the company tries its best to publish academic papers on every study to the greatest extent possible—regardless of whether the study shows negative or positive results.

### Influencing industry standards and regulations

A majority of the companies we studied were engaged in discussions with regulators and industry bodies to encourage the ethical adoption of new science and technology. Some of the smaller firms were working to devise the best method of regulation for an emerging science as demonstrated by Interleukin and Sciona (nutrigenomics) and TGN Biotech (transgenesis to make therapeutic proteins). Others were working with industry groups to encourage the use of high ethical standards in areas of genetic information privacy (as done by Affymetrix), animal testing (Pipeline Biotech), and human rights standards (Novo Nordisk).

### Strategic philanthropy

Philanthropic and drug donation programs are a way for companies to give back to, and engage with, society. The latter strategy tends to be limited to the larger firms that have reached profitability, while smaller firms donate employee time and expertise to address societal needs. Merck has created a nonprofit foundation that has invested hundreds of millions of dollars in public–private partnerships to help build infrastructure and deliver needed drugs in Africa and South America to address HIV/AIDS, and for other health crises, such as river blindness. Another example is Novo Nordisk's World Diabetes Foundation, which supports partnerships and initiatives around the world that help build health infrastructure and health-care capacity in these countries. Novo Nordisk works with local organizations and governments to learn what is needed from the developing country's perspective.

## Approach Five: Ethics Evaluation and Reporting Mechanisms

From our interviews, we found that a few of these companies have methods for evaluating their approach to ethics and for reporting their ethics commitments to stakeholders. Our study originally intended to collect evaluations of each ethics mechanism in order to assess the effectiveness of different approaches. Unfortunately, we found that too few of the companies we interviewed are evaluating their approach to ethics for us to obtain concrete results. However, the following is a description of a few of the evaluation and reporting mechanisms that are starting to be used by some of the larger companies in our study.

Merck now requires that every philanthropic initiative they invest in to be subject to an evaluation process in order to assess whether it truly produced the benefit sought, both for the recipient and for the company. Novo Nordisk has internal ethics auditors who rotate through departments and perform ethics assessments on how well employees are living up to their ethical mandates. These ethics auditors evaluate the department and help devise improvement strategies on an as-needed basis. Both Monsanto and Novo Nordisk have a mechanism in place that reports to the public on their initiatives. Monsanto's Pledge Progress Reports and Novo Nordisk's Sustainability Reports are meant to transparently describe the companies' stances on issues and their efforts to live up to their ethical promises.

Our findings in the area of ethics evaluation demonstrate a need for future development and research. For bioscience companies who are more familiar with tangible and quantitative outcomes (with respect to share price, market share, and scientific data and results), it is challenging to devise a method to evaluate something as intangible as ethics. Employee surveys, public opinion polls, share price, and product acceptance levels were some of the measurement approaches suggested during our interviews. Although many of the companies studied are not evaluating the effectiveness of their ethics mechanisms, it was very clear that companies feel that evaluating their ethics approaches in order to learn from their successes and failures is a vital component of any bioindustry ethics initiative.

## Limitations of Our Approach

The objective of this paper is to highlight specific mechanisms used by companies to address their ethical issues. However, we recognize that the views of senior management of bioscience companies are not the only relevant perspectives on these issues. We feel that one important next step would be to engage the opinions of other key players, such as nongovernmental organizations, governments, academics, and the general public.

Another limitation of a study such as this is the risk of social desirability bias. This occurs when the research participant expresses a viewpoint that he or she thinks the interviewer wants to hear rather than what he or she truly believes. Although management opinions were given in this research study, the mechanisms described in this article are not opinions but rather a description of mechanisms being used by the companies—and, thus, they are less subject to bias. At each company, the descriptions of the mechanisms were given by more than one interviewee, and in most cases, we had documents supporting the fact that these mechanisms do occur as described. We recognize these limitations, but feel that because the people we interviewed are closest to the phenomenon, they represent a legitimate viewpoint and a highly logical entry point for empirical research into why and how bioscience companies address ethical issues.

## Conclusion

Our study uncovered five interrelated approaches, each with several mechanisms to address bioindustry ethics. Based on our findings, a company of any size can start with strong ethical leadership and seek external ethics expertise early on. Internal ethics mechanisms and external ethics engagement mechanisms are other approaches that a bioscience company of any size can implement. As demonstrated by the larger companies in our study, companies can also develop ethics evaluation and reporting mechanisms that aim to keep the company on track and encourage management to monitor the outcomes of their ethical decision making. The mechanisms reported in this article demonstrate ideas for ways in which management in the bioscience industry can begin to address the complex ethical issues facing their companies.

**Box 1. Research Methodology**

Qualitative case study methods were used for this research.

**Data Collection**

Data was collected over a two-year period, using a study design approved by the University of Toronto research ethics board. Our research team performed in-depth, open-ended interviews with managers and executives from 13 bioscience companies (more than 100 interviews in total). Media articles, press releases, and company documents were also analyzed to verify the data resulting from the interviews.

**Data Sources**

Data was drawn from (1) interview notes, (2) observations from company visits, and (3) written documents (produced by the company and by other sources).

**Data Analysis**

Case Studies. The three sources of data were analyzed for each company independently to produce 13 qualitative case studies describing ethical decision making in each company. These case studies were verified for accuracy and approved for publication by each firm.

Cross-Case Comparison. To perform the comparison, the case studies and interview notes were coded on four themes: (1) What mechanisms are bioscience companies using to address their ethical issues? (2) How effective are their mechanisms? (3) Why have these bioscience companies decided to implement ethics mechanisms? (4) What ethical issues are these bioscience companies facing and addressing with the previously mentioned mechanisms?

In qualitative research, this is known as axial coding. This coding process was performed first by one of our researchers and then verified for validity by other team members. Any discrepancies in results were discussed until consensus was reached.

The results from Themes 1 and 2 are discussed in the text of this article, and Themes 3 and 4 are addressed in Box 3 and Table 2, respectively.

**Box 2. BioIndustry Ethics Mechanisms**

Ethical leadership

- Founder/CEO/management ethical leadership
- An ethics department

External expertise

- External ethics consultant
- Ethics advisory boards

Internal ethics mechanisms

- Hiring practices focused on ethics
- Employee performance evaluations
- Ethics education and forums for ethics discussion
- Ethical reinforcement techniques

External ethics engagement

- Ethics mechanisms with partners and suppliers
- Transparent engagement mechanisms with stakeholders
- Transparency of science
- Influencing industry standards and regulations
- Strategic philanthropy

Ethics evaluation and reporting mechanisms

**Box 3. Why Are Bioscience Companies Addressing BioIndustry Ethics?**

- Do the “right thing”
- Risk mitigation
- Public reputation
- Attract and keep the “right” employees
- Guidance in uncharted waters
- Promote good science

## Abbreviation

EAB: Ethics Advisory Board
